# Interrogating the Role of miR-125b and Its 3′isomiRs in Protection against Hypoxia

**DOI:** 10.3390/ijms242116015

**Published:** 2023-11-06

**Authors:** Lee Lee Wong, Azizah Binti Fadzil, Qiying Chen, Miriam T. Rademaker, Christopher J. Charles, Arthur Mark Richards, Peipei Wang

**Affiliations:** 1 Cardiovascular Research Institute, National University Health System, Singapore 117599, Singapore; mdcaziz@nus.edu.sg (A.B.F.); chenqiying202202@163.com (Q.C.); mark.richards@nus.edu.sg (A.M.R.); 2 Department of Medicine, Yong Loo Lin School of Medicine, National University of Singapore, Singapore 117599, Singapore; 3 Christchurch Heart Institute, Department of Medicine, University of Otago-Christchurch, Christchurch P.O. Box 4345, New Zealand; miriam.rademaker@otago.ac.nz

**Keywords:** microRNA, isomiR, miR-125b, hypoxia, and apoptosis

## Abstract

MiR-125b has therapeutic potential in the amelioration of myocardial ischemic injury. MicroRNA isomiRs, with either 5′ or 3′ addition or deletion of nucleotide(s), have been reported from next-generation sequencing data (NGS). However, due to technical challenges, validation and functional studies of isomiRs are few. In this study, we discovered using NGS, four 3′isomiRs of miR-125b, i.e., addition of A (adenosine), along with deletions of A, AG (guanosine) and AGU (uridine) from rat and sheep heart. These findings were validated using RT-qPCR. Comprehensive functional studies were carried out in the H9C2 hypoxia model. After miR-125b, isomiRs of Plus A, Trim A, AG and AGU mimic transfection, the H9C2 cells were subjected to hypoxic challenge. As assessed using cell viability, apoptosis, CCK-8 and LDH release, miR-125b and isomiRs were all protective against hypoxia. However, Plus A and Trim A were more effective than miR-125b, whilst Trim AG and Trim AGU had far weaker effects than miR-125b. Interestingly, both the gene regulation profile and apoptotic gene validation indicated a major overlap among miR-125b, Plus A and Trim A, whilst Trims AG and AGU revealed a different profile compared to miR-125b. Conclusions: miR-125b and its 3′ isomiRs are expressed stably in the heart. miR-125b and isomiRs with addition or deletion of A might function concurrently and concordantly under specific physiological and pathophysiological conditions. In-depth understanding of isomiRs’ metabolism and function will contribute to better miRNA therapeutic drug design.

## 1. Introduction

Myocardial infarction (MI) is a leading cause of heart failure (HF) and ultimately death. MicroRNAs (miRNAs) bind to the 3′untranslated region (UTR) of their mRNA targets and down-regulate genes via mRNA degradation or translational inhibition. MiRNAs are involved in diverse aspects of cardiac pathophysiological processes including hypertrophy, remodeling, HF, and arrhythmia [1,2,3]. Recent reports indicate miRNAs protect the heart against ischemic injury by regulating key signaling elements and are therefore potentially therapeutic targets [4]. miR-125b is highly conserved in mammals. Mature miR-125b, also called miR-125b-5p, is transcribed from two loci located on chromosomes chr11 and chr21 as hsa-miR-125b-1 and -2; chr9 and chr16 as mmu-miR-125b-1, and -2; chr8 and chr11 as rno-miR-125b-1 and -2, respectively [5]. MiR-125b is well-studied since it is implicated in a wide range of physiological and pathological processes. Clinically, miR-125b is down-regulated in the hearts of patients with end-stage dilated cardiomyopathy (DCM) or ischemic cardiomyopathy [6]. Lower circulating levels of miR-125b-5p are associated with an increased occurrence of acute myocardial infarction (AMI). Similarly, miR-125b has been reported to protect hearts from ischemia/reperfusion injury. It was demonstrated that miR-125b mimics reduce apoptosis via direct targeting of Bak1, Bmf, TRAF6 and klf13 [7,8].

Mature miRNAs are small with 20 to 24 nucleotides (nts). They are identified using short-tag RNA-sequencing (RNA-seq). Widespread usage of high-throughput RNA-seq has revealed that multiple variants of mature miRNAs occur in various plant and animal species. These miRNA variants include nucleotide substitutions, insertions or deletions of 1 or more nts. Among them, 5′ and/or 3′ end non-templated additions and cleavage variations are most frequent [9]. Canonical miRNAs and isomiRs are loaded to Argonature (Ago), as detected using co-immunoprecipitation, and bind to the 3′UTRs of specific mRNA targets, as shown using luciferase reporter assay [10,11]. Therefore, isomiRs are potentially functional miRNAs.

The functional significance of isomiRs is not yet well-understood. Such studies are very much limited to 5′ isomiRs as changes to the seed region are associated with altered regulation of target genes [12]. Target prediction indicated 642 potential targets for miR-411 but 1249 targets for the 5′ isomiR (with an addition of adenosine) with 269 overlapping targets. The 5′ isomiR negatively regulated cell migration whilst miR-411 had no such effect [13]. miR-21–5p suppresses growth hormone receptor (GHR) in liver cancer. The up-regulation of 5′ isomiRs with addition or deletion of 1 nt leads to loss of GHR suppression with consequent stimulation of cancer cell growth [14]. Most published NGS results indicate that 5′ isomiRs are rare with 3′ isomiRs predominant [9]. Functional alterations and mechanisms associated with 3′ isomiRs are less well-reported. It is generally believed that 3′ isomiRs may affect miRNA stability and turnover [15,16]. Whether isomiR length variation at 3′ end affects miRNA function is unknown. In one report, miR-222 was anti-apoptotic whilst its 3′ extended isoforms were pro-apoptotic via inhibition of the *PI3K-AKT* pathway [17].

This study demonstrates the cardiac ubiquity of miR-125b and associated 3′ isomiRs with addition of A, along with deletion of A, AG, and AGU in healthy and ischemic rat hearts and healthy and failed sheep hearts. IsomiRs with one added or one deleted A at the 3′ terminal of mir-125b have similar cardioprotective effects to canonical miR-125b-5p. In contrast, isomiRs with 3′ deletions of AG and AGU show loss of action. Our study provides a better understanding of the concurrent involvement of miR-125b and its isomiRs in cardiac-protective processes and may allow for the development of novel therapies for cardioprotection.

## 2. Results

### 2.1. The Overexpression of miR-125b Reduces Cell Injury and Inhibits Apoptosis

**Figure 1:**
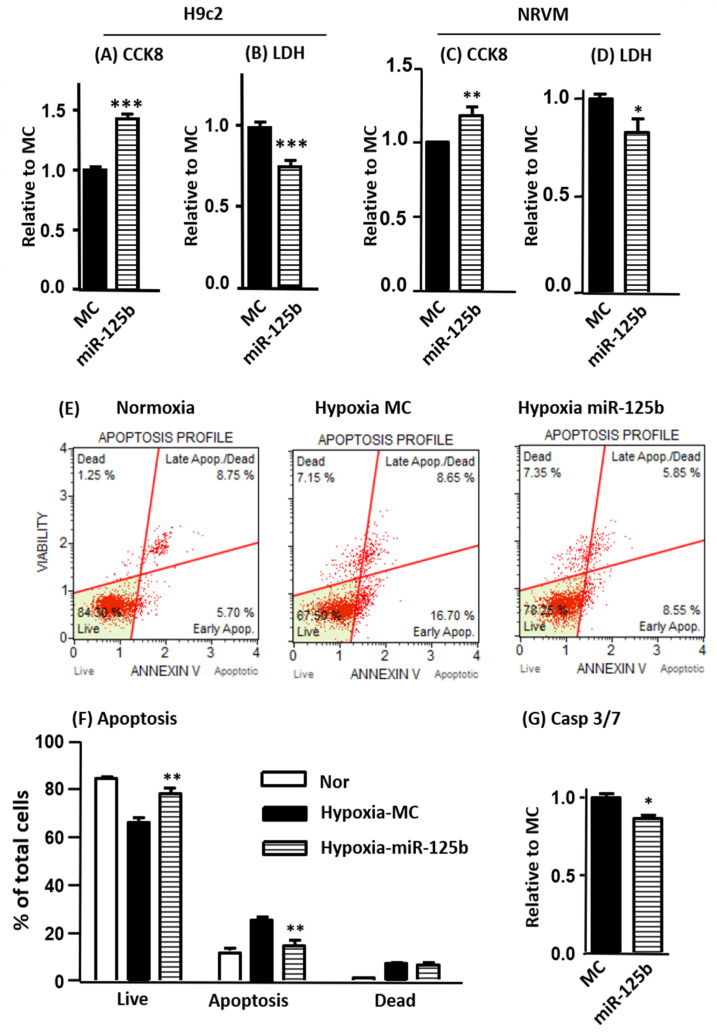
Protective effects of miR-125b in hypoxia model of H9C2 and NRVM. In this study, 25 nM miR-125b mimic or mimic control (miR-125b or MC) was transfected to H9C2 or NRVM. Cells were subjected to 24 h of hypoxia (0.2% oxygen) or normoxia. Cell injury was measured using CCK-8 and LDH release in H9C2 (**A**,**B**) and NRVM (**C**,**D**). Apoptosis was assessed using Annexin V and 7-AAD double staining followed by flow cytometry measurements. Representative apoptosis profiles and calculations are shown for each group (**E**,**F**). The inhibition of apoptosis was further confirmed using significantly reduced caspase3/7 activity (**G**). Data are presented as mean ± SD with Student’s *t*-test, * *p* < 0.05, ** *p* < 0.01, *** *p* < 0.001 vs. MC in two-tailed unpaired *t*-test. Data represent the mean ± SD of triplicate experiments.

After 24 h transfection, H9C2 cells were subjected to 24 h of hypoxia. Our results showed that miR-125b mimics drastically reduced cell injury, as indicated by increased CCK-8 activity and reduced lactate dehydrogenase (LDH) release (Figure 1A,B). Cell apoptosis was determined in H9C2 using ANNEXIN V and 7-ADD (7-amino-actinomycin) staining showing that hypoxia-induced apoptosis and cell death was reduced by miR-125b (Figure 1E,F). The inhibition of apoptosis was further confirmed using significantly reduced caspase3/7 activity (Figure 1G). The cardioprotective effects of miR-125b (increased CCK-8 and reduced LDH release) were replicated in our NRVM hypoxia model (Figure 1C,D).

### 2.2. The Overexpression of miR-125b in H9C2 Cells Down-Regulates Multiple Pro-Apoptotic Genes

**Figure 2:**
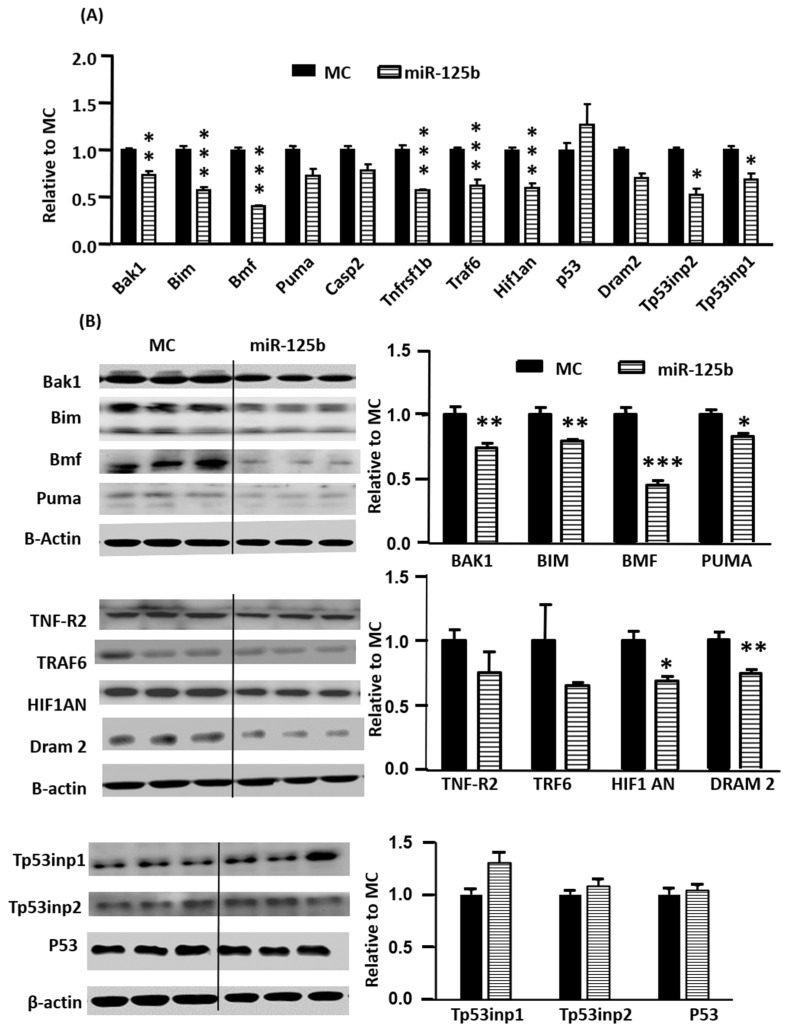
Assessments of miR-125b predicted target expression using RT-qPCR and Western blot in H9C2. MiR-125b regulates the expressions of mRNA *Bak1, Bim, Bmf, Tnfrsf1b, Traf6, Tp53inp1,* and *Tp53inp2* (**A**) and protein levels for BAK1, BIM, BMF, PUMA, HIF1AN, and DRAM 2 (**B**). Data are presented as mean ± SD with Student’s *t*-test, * *p* < 0.05, ** *p* < 0.01, *** *p* < 0.001 vs. MC in two-tailed unpaired *t*-test.

MiR-125b-predicted gene targets in association with apoptosis were selected for assessments using RT-qPCR and Western blot. miR-125b mimics significantly inhibited the mRNA expression of *Bak1*, *Bim*, *Bmf*, *Tnfrsf1b* (protein name TNF-R2), *Hif1an* (protein name HIF-1), *Traf6*, *Tp53Inp2* and *Tp53Inp1* (Figure 2A). For protein levels, only BAK1, BIM, BMF, PUMA, HIF1AN and DRAM2 were significantly decreased by miR-125b (Figure 2B). The mRNA expressions of *Tp53inp1* and *Tp53inp2* were decreased by miR-125b but levels of the proteins coded by these genes were unchanged.

To further validate whether miR-125b directly targets the 3′UTR of these genes, we constructed target gene 3′UTR cDNA luciferase reporters and co-transfected with miR-125b mimic. A luciferase activity assay was performed 24 h after transfection (Supplementary Figure S1). The luciferase activities from the reporters containing the 3′UTR of *Bak1*, *Bmf*, *Puma*, *Tnfrsf1b*, *Dram2* and *Tp53inp2* were significantly decreased by miR-125b, indicating direct binding to 3′UTR. There was no effect on *Traf6* or *p53* 3′UTR.

### 2.3. The Assessment of miR-125b isomiRs Using NGS and Validation Using RT-qPCR

**Figure 3:**
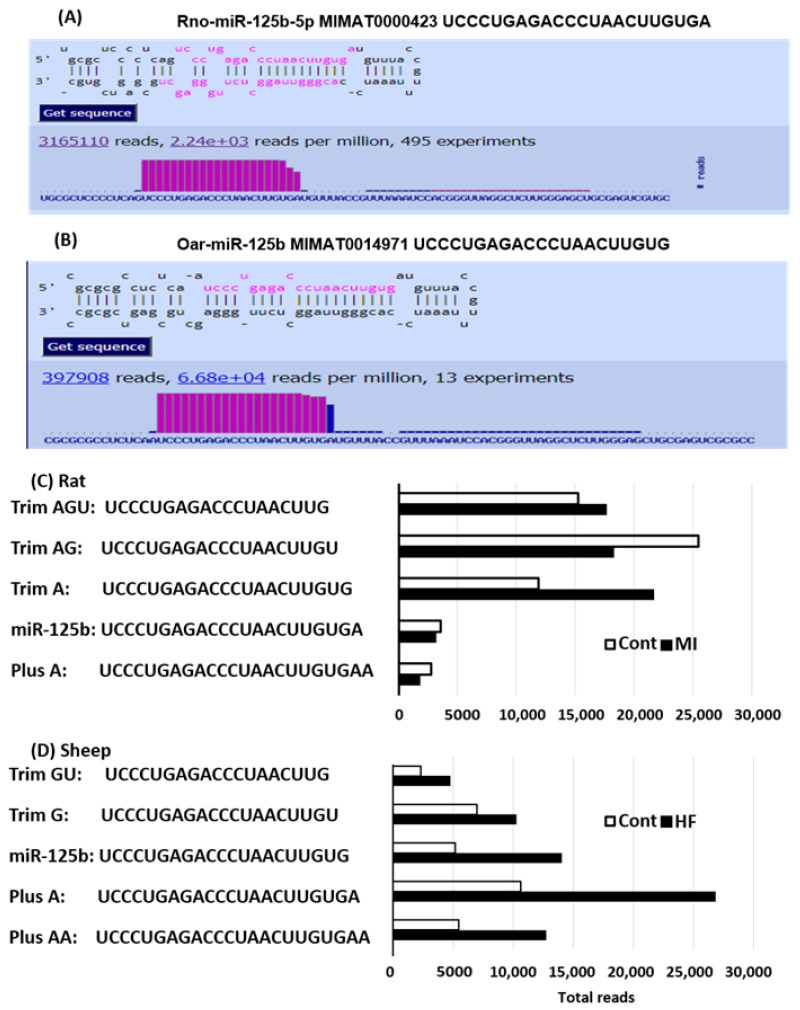
Detection of miR-125b and its isomiRs in the healthy and diseased rat and sheep hearts. Hairpin structures for rno-miR-125b (rat) and oar-miR-125b (sheep) from miRbase are presented in (**A**,**B**), respectively. The expressions of miR-125b and its isomiRs were assessed using next-generation sequencing (NGS). MiR-125b and isomiRs were detected in the rat healthy and MI hearts (**C**) and in the sheep healthy and HF hearts (**D**).

The miRbase miR-125b hairpin structure and sequences for rats and sheep are illustrated in Figure 3A,B, respectively. The canonical oar-miR-125b appears to be the isoform for rno-miR-125b with a deletion of A at the 3′ end. Heart samples from a rat ischemia model and ovine rapid ventricular pacing-induced HF model [18,19] were collected for miRNA sequencing. miR-125b and its four isoforms were highly expressed in both models (Figure 3C,D). The top five expressed miR-125b and its isomiRs in both rat and ovine datasets were the same regardless of the canonical sequence differences.

**Figure 4:**
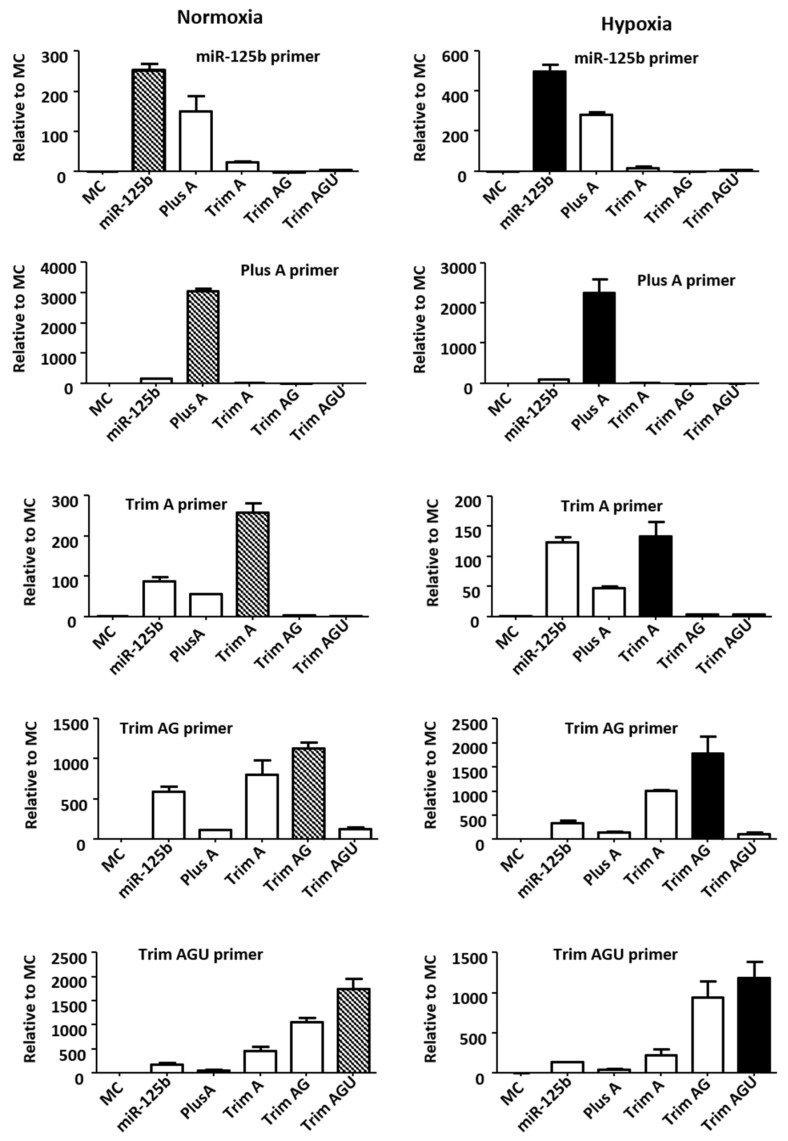
Measurements of miR-125b and its isomiRs using RT-qPCR. In this study, 25 nM mimics of miR-125b or isomiRs of Plus A, Trim A, Trim AG, Trim AGU or MC were transfected to H9c2 followed by 24 h of normoxia or hypoxia (0.2% oxygen) culture. Specific primers were used to validate the transfections. Fold changes relative to MC are presented. Data are presented as mean ± SD of triplicate experiments.

IsomiRs have been frequently reported using NGS technology. Due to the minor sequence difference, they are not well-validated using RT-qPCR. For this, we engaged Genepharma Co. Ltd. (Shanghai, China) to design specific primers targeting miR-125b and its isoforms. In H9C2 cells transfected with miR-125b and isomiRs under normoxic and hypoxic conditions, the primers were highly specific, as validated using qPCR and confirmed using Sanger sequencing (Supplementary Figure S2). Among them, Plus A had the best specificity (Figure 4).

### 2.4. miR-125b and isomiRs Are Protective but Trim AG and Trim AGU Are Less Effective than Others

Next, we compared the function of miR-125b and isomiRs in a comprehensive way. Under normoxic conditions, they did not affect cell viability (Supplementary Figure S3A), CCK-8 (Supplementary Figure S3B), or apoptosis, as detected using ANNEXIN V and 7-AAD double staining (Supplementary Figure S3C).

**Figure 5:**
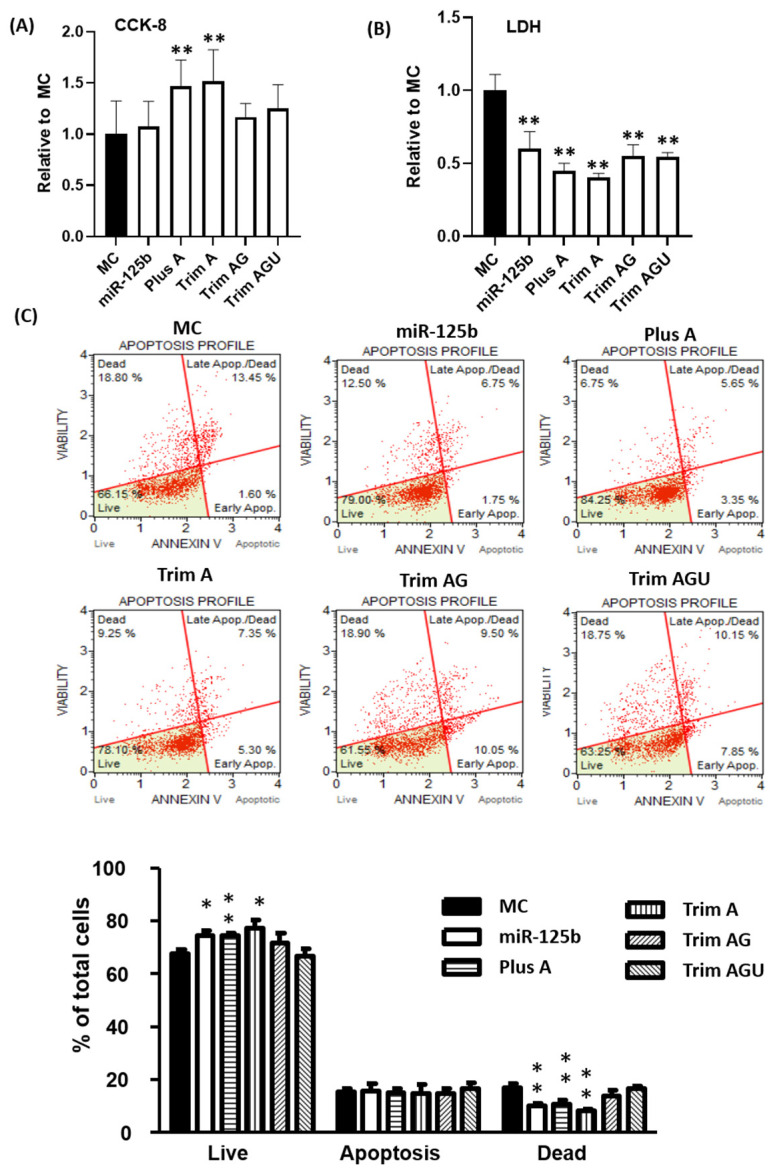
Protective effects of miR-125b and its isomiRs. In this study, 25 nM mimics of miR-125b or isomiRs of Plus A, Trim A, Trim AG, Trim AGU or MC were transfected to H9C2. Cells were subjected to 24 h of hypoxia (0.2% oxygen). Cell injury was measured using CCK-8 (**A**) and LDH release (**B**). Apoptosis was assessed using Annexin V and 7-AAD double staining followed by flow cytometry measurements (**C**). Representative apoptosis profiles and calculations are shown for each group (**C**). Data are presented as mean ± SD with Student’s *t*-test, * *p* < 0.05, ** *p* < 0.01 vs. MC in two-tailed unpaired *t*-test. Data are presented as mean ± SD of triplicate experiments.

The protective effects under hypoxia were evaluated using the same approach. miR-125b and isomiRs increased cell viability and reduced iLDH release (Figure 5B), although Trim AG seemed less potent (Supplementary Figure S4). However, both Plus A and Trim A provided stronger protective effects, as assessed using both CCK-8 and LDH release (Figure 5A,B). The differences among groups were even more significant as assessed using ANNEXIN V and 7-AAD double staining. MiR-125b, Plus A and Trim A exhibited protective effects with an increase in live cells and decrease in dead cells as compared with MC; Trim AG and Trim AGU had no protective effects (Figure 5C). With all functional assessments considered, miR-125b was protective, as is well-documented. However, isomiRs of Plus A and Trim A showed elevated protective effects compared to miR-125b, while Trim AGU and Trim AG had weaker effects.

### 2.5. Profiling of miR-125b and isomiRs Induced Gene Regulation under Normoxic and Hypoxic Conditions

**Figure 6:**
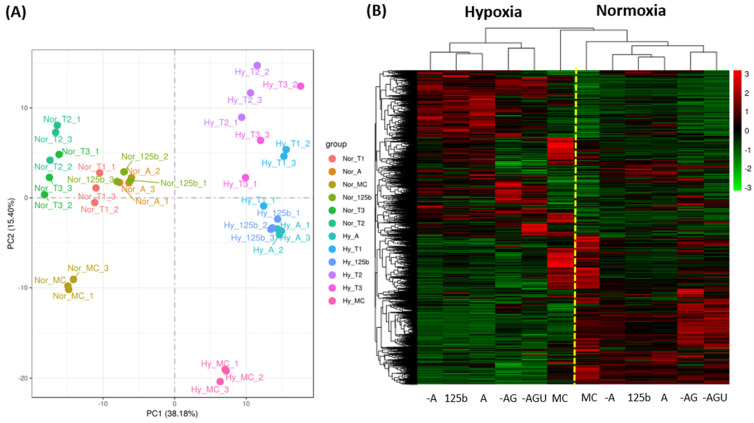
The profiles of gene regulation by miR-125b and its isomiRs in H9C2 under normoxic and hypoxic conditions. In this study, 25 nM mimics of miR-125b or isomiRs of Plus A, Trim A, Trim AG, Trim AGU or MC were transfected to H9C2. Cells were subjected to 24 h of normoxia and hypoxia (0.2% oxygen). Gene expression levels were assessed using NGS. The profiles for the regulation of gene expression were visualized using a PCA plot (Nor: Normoxia, Hy: Hypoxia, T1: Trim A, T2: Trim AG and T3: Trim AGU) (**A**) and hierarchical clustering heat map based on mean gene expression (N = 3 for each group) (**B**).

RNA sequencing was performed in triplicate for each group. More than 30,000 genes were detected using NGS. The PCA plot shows a distinct separation between normoxia and hypoxia (Figure 6A). Notably, MC groups were separated from miR-125b and isomiR groups under both conditions. The changes in gene profiling using miR-125b, Plus A and Trim A transfection were closely matched. Changes induced by Trim AG and Trim AGU were more profound than those changes in miR-125b, Plus A and Trim A. These differences were distinct in the hierarchical clustering heat map (Figure 6B). Individual treatment results are included in Supplementary Figure S5.

**Figure 7:**
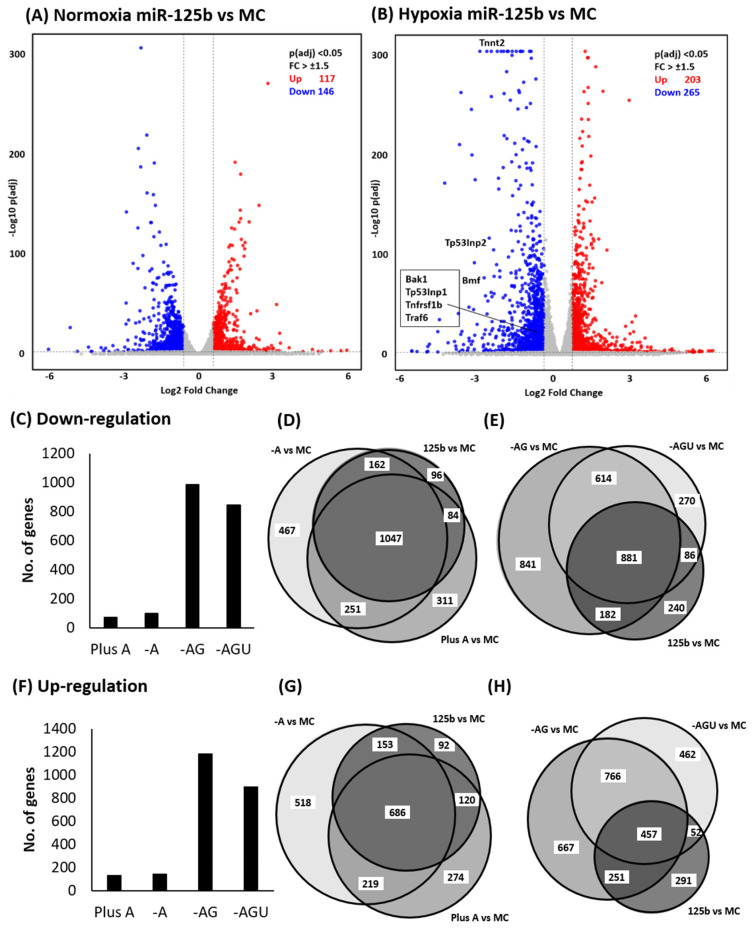
The differences in gene regulation by miR-125b and its isomiRs in H9C2 under normoxia and hypoxia. In this study, 25 nM mimics of miR-125b or isomiRs of Plus A, Trim A, Trim AG, Trim AGU or MC were transfected to H9C2. Cells were subjected to 24 h of normoxia and hypoxia (0.2% oxygen). Gene expression levels were assessed using NGS. Up—(red) and down—(blue)-regulated genes with ≥±1.5-fold change, p(adj) < 0.05, in miR-125b vs. MC, are presented in volcano plots: (**A**) normoxia and (**B**) hypoxia. The numbers of genes that are differently regulated by isomiRs compared to miR-125b under hypoxic conditions are indicated in (**C**) for down-regulation and (**F**) up-regulation. The overlapping gene regulations compared to MC between miR-125b and its isomiRs are presented in the proportional Venn diagram, (**D**,**E**) for down-regulation of miR-125b, Plus A and Trim A with miR-125b, Trim AG and Trim AGU, respectively. (**G**,**H**) Up-regulated genes presented with a similar arrangement.

Next, we examined those genes significantly regulated by miR-125b and isomiRs, as indicated by ≥1.5-fold change with p(adj) < 0.05 compared to MC. The volcano plots showed 146 and 265 genes down-regulated by miR-125b vs. MC in normoxia and hypoxia, respectively (Figure 7A,B). In total, 117 and 203 genes were up-regulated by miR-125b in normoxia and hypoxia, respectively. To understand the differences in gene regulation between miR-125b and its isomiRs, we directly compared up- and down-regulated genes between miR-125b and isomiRs. Most of the genes down-regulated by Plus A and Trim A were overlapped with miR-125b, with less than 100 genes differentially down-regulated. However, the genes regulated by Trim AG and Trim AGU were remarkably different from miR-125b with 10 times more than those in Plus A and Trim A (Figure 7C). The number of genes significantly down-regulated by miR-125b and isomiRs is further illustrated in Figure 7D,E. The comparison of up-regulated genes between miR-125b and isomRs shows a similar pattern (Figure 7F–H). Additional analysis of gene regulation under normoxia gave a similar finding (Supplementary Figure S6).

### 2.6. Gene Expression Validation Using RT-qPCR

**Figure 8:**
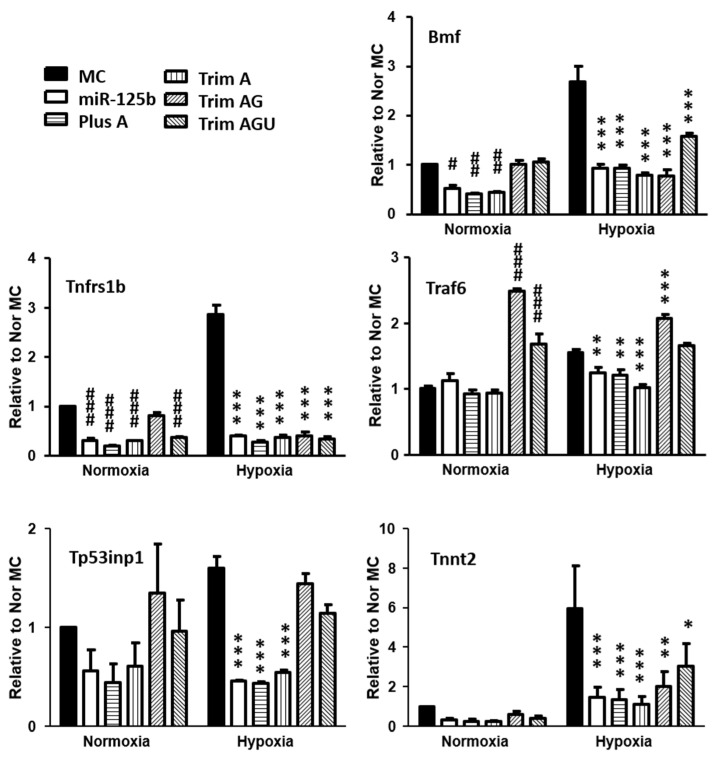
Gene expression for miR-125b targets was assessed using RT-qPCR. In this study, 25 nM mimics of miR-125b or isomiRs of Plus A, Trim A, Trim AG, Trim AGU or MC were transfected to H9C2. Cells were subjected to 24 h of normoxia and hypoxia (0.2% oxygen). Selected target gene regulations were validated using RT-qPCR. Data are presented as mean ± SD with Student’s *t*-test, # *p* < 0.05, ## *p* < 0.01, ### *p* < 0.001 vs. mormoxia MC, * *p* < 0.05, ** *p* < 0.01, *** *p* < 0.001 vs. hypoxia MC (two-tailed).

The most down-regulated gene by NGS, *Tnnt2*, and some predicted miR-125b apoptotic targets were selected for further validation. The apoptosis cofactor, *Bmf*; the TNF induced apoptosis gene, *Tnfrs1b*; the positive gene regulation for autophagy cell death gene, *Tp53inp 1*; and *Traf 6* and *Tnnt 2* were significantly down-regulated by miR-125b and its isomiRs (Figure 8). Interestingly, the validation of gene expression supported the finding from NGS where miR-125b, Plus A and Trim A exerted similar effects on the target gene regulation, while Trim AG and Trim AGU yielded different effects (Figure 8 and Supplementary Figure S7).

## 3. Discussion

miR-125b is preserved across species and has been well-documented to promote cell survival through its anti-apoptotic function. miR-125b plays a role in the development of cardiovascular disease including myocardial ischemia, atherosclerosis, ischemia–reperfusion injury, ischemia stroke and HF [20]. Due to its multifaceted functions, this makes miR-125b an interesting therapeutic target in cardiovascular disease. With miR-125b mimic transfection in an H9C2 hypoxic model, we confirmed that overexpression of miR-125b promotes cell survival with enhanced CCK-8 cell viability and decreased LDH cell injury levels. For the first time, we now demonstrate the high abundance of miR-125b and it 3′ isomiRs with addition of A, along with deletions of A, AG, and AGU in healthy and ischemic rat hearts and healthy and HF sheep hearts. In functional studies, we showed that 3′ isomiRs with additional A and Trim A at 3′ end have similar or even enhanced protective effects compared to canonical miR-125b. In contrast, the protective effects of isomiRs Trim AG and Trim AGU are reduced as compared to canonical miR-125b.

The first miRNA *Lin-4* was discovered from *Caenorhabditis elegans* in 1984 [21]. *Lin-4* is the first-discovered miRNA with 22 nucleotides. It does not encode a protein but directly binds to *Lin-14* 3′UTR and down-regulates LIN-14 protein expression, which plays an important role in the maturation of *C. elegans* [22]. Later on, the *Lin-4* ortholog, miR-125b, was discovered in mammals. miR-125b is highly preserved across species and plays an important role in neuronal differentiation [23]. miR-125b is multi-functional, acting as a tumor suppressor or an oncogene depending on the cellular context. MiR-125b promotes proliferation and migration of type II endometrial carcinoma cells and tumor metastasis in patients with non-small-cell lung cancer, by targeting the tumor suppressor TP53INP1 [24]. Up-regulation of miR-125b is cardioprotective via inhibition of ischemia-induced up-regulation of *p53, Bak-1* and *Bax* [7]. BMF and PUMA belong to BH3-only proteins, which are the pro-apoptotic members of the bcl-2 family. Upon activation, they initiate apoptosis by binding and neutralizing Bcl-2 pro-survival proteins via their BH3 domain [25]. BAK1 is required for BH3-only protein cell killing and is an important effector inducing cell apoptosis. miR-125b exerts its pro-survival effect via inhibition of expression of the pro-apoptotic proteins BMF, Puma and BAK1. TNFR2 enhances TNF-α-mediated apoptosis [26]. In this study, we first replicated miR-125b-induced protection in a H9C2 hypoxia model and confirmed that in NRVM. Our results are consistent with previous reports. We showed that miR-125b mimics promote the cell survival and decrease cell death. The protective effect is related to the cell apoptosis pathway by targeting multiple apoptosis signaling genes: *Bak1, Bmf, Bim, Puma, Tnfrsf1b, Traf6*, etc.

Accumulating results from NGS studies demonstrate that isomiRs, e.g., miRNAs with 1–3 nucleotide differences, are generally present [9]. Mature miRNAs are produced from the pri-miRNA stem loop and pre-miRNA hairpins as a result of Drosha in the nucleus and Dicer cleavage in the cytosol, respectively [27,28]. The imprecision of such cleavage is an important mechanism underpinning production of isomiRs. We have detected miR-125b and isomiRs in two different species, rat and sheep, including 3′-trimmed A, AG, and AGU, which are reasonably presumed to be the products of imprecise cleavage. They are classified as templated as these sequences originate from the same hairpin genes. An isomiR with additional A (Plus A) compared to canonical miR-125b was detected, as well. This isomiR can be attributed to the post-transcriptional processing by nucleotidyl transferases [29]. As the additional nucleotide does not exist in the parental hairpin genes, these variants are classified as non-templated. No miR-125b 5′ isomiRs were detected.

It is interesting to point out that the rat canonical miR-125b sequence is identical to hsa-/mmu-miR-125b, and is only one nucleotide different in sheep, i.e., with or without A, according to miRbase 22.1 (http//www.mirbase.org, accessed on 5 December 2022). By definition, canonical miRNA is the predominantly expressed miRNA with the most abundant sequence reads among variant sequences deposited into miRbase by different research groups. In this case, data from only 13 sheep experiments have been deposited vs. 495 rat experiments from sequencing datasets. Therefore, the canonical sequence of oar-miR-125b remains to be confirmed. Our NGS results show that the most enriched oar-miR-125b isoform (Plus A) is the same sequence as rno-miR-125b. More confirmatory results from different studies are required. Notably, isoforms with trimmed A, AG, and AGU are more abundant than canonical miR-125b in rats. Sequence-oriented isomiR annotation (CASMIR) for unbiased identification of global isomiRs indicates that specific isomiRs are often more abundant than their canonical forms [30]. Many reports confirm canonical miRNAs are not always the most enriched isoforms [31,32].

Accumulating reports indicate isomiRs are commonly expressed, and the tissue isomiR profiles are cell-type-specific and context-dependent [12,33]. Functional studies are rare and mostly limited to 5′ isomiRs [34]. As 5′ variants could change the miRNA seed region, they cause target shifting and lead to functional change [13,14,34]. However, 5′ isomiRs only represent a small proportion of isomiRs compared to 3′ isomiRs [9,33]. A study in human brain samples revealed 80–90% of miRNAs have isomiRs, predominantly 3′ trimmings or additions [35]. Furthermore, 3′ isomiRs share the same seed regions as the canonical miRNA, but it is unclear whether they induce similar functions. For the first time we directly compared the protective effects of miR-125b among its four most abundant isomiRs. Our results demonstrate that miR-125b and all its isomiRs are protective, as evaluated via increased cell viability and reduced cell injury in the context of hypoxic challenge. However, anti-apoptotic effects differ between the isomiRs. Of those, Plus A and Trim A perform better than miR-125b, while Trim AG and Trim AGU have much weaker protection. All three of them may well share the same underlying mechanisms as their regulations on gene expression profiles and the pattern of apoptotic gene validation are very similar. Our results also suggest that Plus A and Trim A are more effective than the mature form of miR-125b.

The majority of miRNA isomiRs comprise 3′ trimmings or additions with a nucleotide tail of A or U [36,37]. U-addition may inhibit miRNA activity as evidenced by miR-26b in human adenocarcinoma cells [38]. Recently, a very interesting study demonstrated that miR-27 with 3′ U-addition alters miRNA function by modulating gene target recognition. They named this mechanism Tail-U-Mediated-Repression (TUMR) [39]. It is reported that miRNA with U-addition promotes a group of miRNA degradation after T-cell activation [37]. Whether miRNA A-addition increases or decreases miRNA turnover is unknown. To our knowledge, no functional study of A-addition has been reported. We observed that isomiRs of Plus A and Trim A function similarly as the canonical miR-125b and are even more protective than the canonical form. In this study, we also showed that two isomiRs with further trimming, namely Trim AG and Trim AGU, decreased anti-apoptotic potency. It is believed that miRNA 3′ modification affects miRNA turnover [15]. The progressive trimming might be a mechanism of miRNA degradation. This warrants further investigation.

In conclusion, we discovered four 3′ isomiRs of miR-125b, Plus A, Trim A, Trim AG and Trim AGU, from rat and sheep hearts using NGS. miR-125b and isomiRs are all protective against hypoxia. However, Plus A and Trim A are more effective than miR-125b, while Trim AG and Trim AGU have much weaker effects than miR-125b. Both gene regulation profiling and apoptotic gene validation indicate a wide overlap among miR-125b, Plus A and Trim A. From a therapeutic drug design point of view, it is worthwhile to investigate the effects of different isomiRs to find the most effective one. Possibly pairing isomiRs along with canonical miRNA might function better than a single miRNA under physiological and pathophysiological conditions. In-depth understanding of isomiR metabolism and function may assist better therapeutic design of miRNA drugs.

## 4. Materials and Methods

### 4.1. H9C2 Cell Culture, NRVM Isolation, miRNA Mimic Transfection and Hypoxia Treatment

H9C2 cells (a rat cardiac myoblast cell line) were purchased from American Type Culture Collection (Manassas, VA, USA) and cultured in Dulbecco’s modified Eagle’s medium (DMEM) supplemented with 10% (vol/vol) fetal bovine serum, 2 mM glutamine, 100 units/mL penicillin, and 100 g/mL streptomycin at 37 °C in 5% CO_2_. At 70% confluence, 25 nmol/L Mimic control (MC), miR-125b mimic (miR-125b) or isomiRs including addition of A (Plus A), deletion of A (Trim A), deletion of AG (Trim AG), and deletion of AGU (Trim AGU) were transfected according to the manufacturer’s instructions (GenePharma, Shanghai, China). The transfection complex was formed in antibiotic-free DMEM by incubating MC, miR-125b or isomiR mimics with Lipofactamine^®^ RNAimax (Thermo Fisher Scientific Inc., Singapore) at a ratio of 20 pmol:1 µL for 10 min. The transfection complex was added to the cells and incubated for 24 h.

Following 24 h of transfection, cells were subjected to hypoxia at <0.2% O_2_ in serum-free low-glucose DMEM for 24 h. Control normoxic (Nor) cells were cultured under normal conditions with fresh DMEM. At the end of hypoxia, cell culture medium was collected for measurement of lactate dehydrogenase (LDH) release (TOX7, Sigma-Aldrich, St. Louis, MO, USA), an indicator of cell injury, and cells were counted (cell counting kit-8; CCK-8, Sigma-Aldrich, St. Louis, MO, USA) [40]. Separate H9C2 was used for assessment of apoptosis, extraction of RNA, and measurement of protein, respectively.

Neonatal rat ventricular myocyte (NRVM) was isolated from 1–3-day-old neonatal rats via enzymatic digestion and mechanical dissociation, as reported previously [25]. Purified cardiomyocytes were cultured in DMEM supplemented with 15% (vol/vol) fetal bovine serum, 100 units/mL penicillin, and 100 g/mL streptomycin at 37 °C in 5% CO_2_. The NRVM cells were then subjected to miR-125b mimic transfection and hypoxic challenge, as described as above.

### 4.2. Cell Injury Assessments with Lactate Dehydrogenase Release, CCK-8, and Apoptosis

Hypoxia culture medium was collected and centrifuged at 250 g for 5 min. The LDH assay mixture was prepared according to the manufacturer’s protocol. A 1:2 volume of mixture and sample supernatant was mixed and incubated at room temperature for 30 min. The reaction was terminated by adding 1/10 volume of 1 N HCl. The absorbance was measured at 490 nm. Finally, 10 μL of the CCK-8 solution was added to H9C2 at the end of normoxia and hypoxia, and then incubated for 4 h. Absorbance at 450 nm was measured.

H9C2 was trypsinized at the end of normoxia and hypoxia. Cell viability and apoptosis were assessed usingANNEXIN V and 7-AAD staining following the manufacturer’s protocol using the MUSE^TM^ Cell Analyzer (Merck Millipore, Singapore) [26]. Based on the staining, cells were separated into 4 sub-groups (ANNEXIN V and 7-AAD double negative, ANNEXIN V only positive, ANNEXIN V/7-AAD double positive, and 7-AAD only positive) corresponding to viable, early-apoptotic, late-apoptotic, and dead cells, respectively.

### 4.3. In Vivo Model

The surgical preparation and study protocol for the ovine rapid ventricular pacing-induced HF model were published previously [18]. The ovine study protocol was approved by the Animal Ethics Committee of the University of Otago, Christchurch, New Zealand. The rat study protocol was approved by the Institutional Animal Care and Use Committee of the National University of Singapore and complied with the Guide for the Care and Use of Laboratory Animals published by the National Institutes of Health (NIH publication no. 85–23, revised 1996). All methods were carefully performed according to the guidelines of the Declaration of Helsinki. For the in vivo rat MI model, the rats were anesthetized with ketamine and xylazine (75 and 10 mg/kg, respectively, intraperitoneally [i.p.]), intubated, and ventilated. The chest was opened with an incision between the left third and fourth ribs. The left anterior descending coronary artery (LAD) was ligated using a 7/0 polypropylene suture. The chest was closed. An identical surgical procedure without LAD ligation was carried out to generate a control group.

### 4.4. miRNA and mRNA Sequencing

Next-generation deep sequencing and RNA sequencing were used to detect miRNA and mRNA, respectively. Total RNA from H9C2 was extracted using TRIzol reagent (Invitrogen, Thermo Fisher Scientific Inc., Singapore) followed by DNase I treatment. RNA quality was determined using a Nanodrop and Bioanalyzer. A total of 100 ng RNA from the control, HF and MI from rat and sheep samples was converted into small RNA libraries according to the Illumina^®^ manufacturer’s instructions. The libraries were assembled and sequenced on the Illumina NextSeq 500 system (Illumina Inc., San Diago, CA, USA). The raw NGS reads were subjected to data quality control via measurement of the quality score (Q-score, a prediction of the probability of an incorrect base-call) and filtered to remove background reads of <15 nucleotides (nts). The sequences with lengths between 15 and 30 nts were analyzed. The sequence analyzing pipeline was carried out by performing alignment with known miRNAs in miRBase v22.1 with Blast, followed by mapping to the respective genome species.

The RNA sequencing was performed using the non-stranded poly-A library method. Briefly, mRNA was purified from total RNA using poly-T oligo-attached magnetic beads. After fragmentation, the first-strand cDNA was synthesized using random hexamer primers, followed by the second-strand cDNA synthesis using dTTP for the non-directional library. This was followed by library construction. The library was sequenced on Illumina platforms, according to effective library concentration and data amount. The sequencing was performed by Novogene AIT, Singapore.

### 4.5. Analysis of miR-125b isomiRs

Sequencing reads were mapped to the species genome and miRbase v22.1. Sequences that mapped to miR-125b were analyzed for their isomiR modifications, namely 5′ trimming, 3′ trimming, nucleotide substitution and 3′ addition. The isomiRs’ distribution at the 3′ ends for miR-125b were compared among the two sequencing datasets obtained from the rat and sheep models.

### 4.6. Real-Time qPCR to Detect and Quantify miRNAs, isomiRs and mRNAs

The miRNA and isomiR expression were determined via hairpin reverse transcription (RT)-qPCR using isomiR-specific RT and qPCR primers (Shanghai GenePharma Co. Ltd., Shanghai, China) and the SuperScript™ IV First-Strand Synthesis System (Invitrogen, Life Technologies, Thermo Fisher Scientific Inc., Singapore). The resulting cDNA was diluted 20× and used as template for qPCR using the SensiFAST™ SYBR^®^ Lo-ROX Kit (Bioline, Meridian Bioscience, Memphis, TN, USA). The data were normalized to U6 as the reference gene.

Messenger RNA expression was determined via universal RT using the SensiFAST™ cDNA Synthesis Kit (Bioline, Meridian Bioscience, Memphis, TN, USA). The cDNA was diluted 10× and used as template for qPCR using the iTaq Universal SYBR Green Supermix (Bio-Rad Laboratories Pte. Ltd., Singapore). Data were normalized to β-actin as the reference gene. All protocols were performed according to manufacturers’ instructions and conducted on the QuantStudio™ 5 Real-Time PCR System (Applied Biosystems™, Thermo Fisher Scientific Inc., Singapore). The miRNA, isomiR and mRNA expression levels were analyzed using the 2^(-delta-delta Ct) method. The forward and reverse primer sequences are summarized in Table 1.

### 4.7. Western Blot

Cells were lysed and quantified according to protein measurement (Bio-Rad Laboratories Pte Ltd., Singapore). Samples of 20 μg protein were loaded to 5–14% SDS-PAGE gels for electrophoresis and then transferred to PVDF membranes overnight at 30V. The following antibodies were used: BMF, HIFa (GeneTex, Axil Scientific, Singapore), β-actin, t-TP53, BAK1, PUMA, DRAM2, TRAF6 (Santa Cruz Biotechnology, Axil Scientific, Singapore), Tp53INP1, TNF-R2 (Sigma-Aldrich, Singapore), and BIM (Cell Signaling Technology, Research Biolabs, Singapore). Bound antibodies were revealed using horseradish peroxidase (HRP)-labeled secondary antibodies (Santa Cruz, Cell Science Pte Ltd., Singapore) and visualized with enhanced chemiluminescence (ECL) detection reagents (Thermo scientific, Singapore).

### 4.8. Statistics

All values are presented as mean ± SD. For RNA sequencing, differential expression analysis between the groups was performed using DESeq2R package (1.20.0). The resulting *p*-values were adjusted using the Benjamini and Hochberg approach for controlling the false discovery rate. Genes with an adjusted *p*-value ≤ 0.05 found with DESeq2 were assigned as differentially expressed. A statistical package and Graph Pad Prism (San Diego, CA, USA) were used to perform statistical analysis. The proportional Venn diagrams were created using the DeepVenn web application. A *p*-value of less than 0.05 was considered statistically significant. Data were compared for differences using one-way ANOVA followed by Bonferroni post hoc analysis or the unpaired two-tailed *t*-test, as appropriate. Multiple-group comparisons were analyzed using two-way ANOVA followed by Bonferroni post hoc analysis.

## Figures and Tables

**Table 1:** Forward and reverse primer sequences.

IsomiR RT-qPCR Primer Sequences		
IsomiR	Forward Sequence (5′–3′)	Reverse Sequence (5′–3′)
U6	CAGCACATATACTAAAATTGGAACG	ACGAATTTGCGTGTCATCC
hsa-miR-125b-5p mimic	ACTGATAAATCCCTGAGACCCTAAC	TATGGTTTTGACGACTGTGTGAT
hsa-miR-125b-5p mimic A 3prime	TGCGTCCCTGAGACCCTAACT	TATGGTTGTTGACGACTGGTTGAC
hsa-miR-125b-5p mimic trim1	AATCGCTCCCTGAGACCCTAA	TATGGTTGTTGACGACTGGTTGAC
hsa-miR-125b-5p mimic trim2	CAGCGTTCCCTGAGACCCTA	TATGGTTGTTGACGACTGGTTGAC
hsa-miR-125b-5p mimic trim3	CTACGGAATCCCTGAGACCCT	TATGGTTGTTGACGACTGGTTGAC
**mRNA RT-qPCR primer sequences**		
**Gene**	**Forward sequence**	**Reverse sequence**
β-actin	GTACAACCTTCTTGCAGCTCCTC	TGACCCATACCCACCATCAC
Bak1	GCCTACGAACTCTTCACCAAG	CACGCTGGTAGACATACAGG
Bim	AGACGAGTTCAATGAGACTTACAC	CGGAAGATGAATCGTAACAGTTG
Bmf	TTGCAGACCAGTTCCATCG	CCCTTCCCTGTTTTCTCGTC
Traf6	CCTCATAAGAGAACAGATGCCT	CGTGCCAAGTGATTCCTCT
HIF1an	ACATTGAGAAGATGCTTGGAGAG	CATGTGGACAGGATAGCAGTC
Tnfrsf1b	PrimeTime^®^ qPCR Primer Assay (Assay ID: Rn.PT.58.35970749; Integrated DNA Technologies, Inc., Singapore)	
Tp53	PrimeTime^®^ qPCR Primer Assay (Assay ID: Rn.PT.58.35888325; Integrated DNA Technologies, Inc., Singapore)	
Tp53inp1	TCCTGGTCTCAGTGAAGCTA	ACAGCAGTGAATGTGCGT
Tp53inp2	CACCTTCCCCTCACCCT	CCTTCTCCAGCAGCACAG
Dram2	ATTGCCTTACATCAGCGACA	GGTTCTCTTCAGGGTTCAGTG
Puma	GCAGTACGAGCGGCGGAGACAAGAAGAGC	CCCTGGGTAAGGGGAGGAGTCCCATGAAGAG
Casp2	GTCCAAGTCTACAGAACAAACCA	CAGCATCACTCTCCTCACATC
Tnnt2	CGAGCAGCAGCGTATTCGC	CAGCCTTCCTCCTGTTCTCCTC

## Data Availability

The data that support the findings of this study are available in the methods and/or supplementary material of this article. The miRNA and RNA sequencing data were deposited in the Gene Expression Omnibus (GEO) at accession numbers GSE87468 and GSE227958, respectively.

## Supplementary Materials

The following supporting information can be downloaded at: https://www.mdpi.com/article/10.3390/ijms242116015/s1.
