# Supplementary material for: Interrogating the Role of miR-125b and Its 3′isomiRs in Protection against Hypoxia
Source: Int J Mol Sci. 2023 Nov 6;24(21):16015. doi: 10.3390/ijms242116015 (PMC10650460; doi:10.3390/ijms242116015)
Supplement: Supplementary file 1 [file ijms-24-16015-s001.zip › ijms-2688143-SI.pdf]

# SUPPLEMENTARY DATA

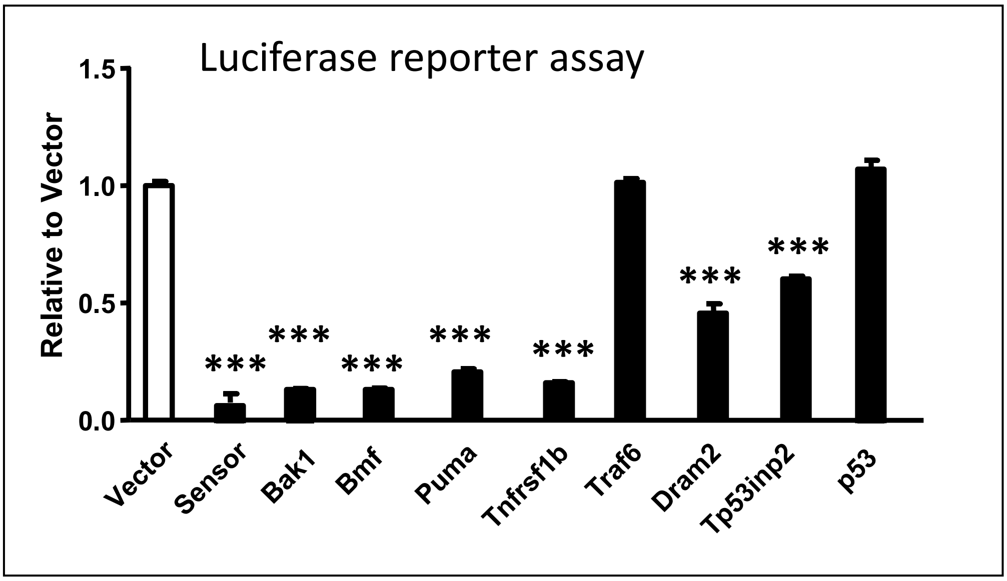


**Suppl. Figure S1:** Confirmation of miR-125b target genes by luciferase reporter assays. Target gene 3'-UTR cDNA sequences were cloned into psiCHECK™-2 dual luciferase reporter plasmid (Promega Corp., Singapore). MiR-125b sensor, the complementary binding sequence to miR-125b, was cloned as a positive control. The plasmids were co-transfected with miR-125b for 24 hr. Renilla and Firefly luciferase activities were measured using Dual-Glo® luciferase assay system (Promega Corp., Singapore). Luciferase activity for miR-125b targets were significantly decreased. Data are presented as Mean ± SD, ***$p < 0.001$ vs. Empty vector by two-tail unpaired t-test. N=3 in triplicates.

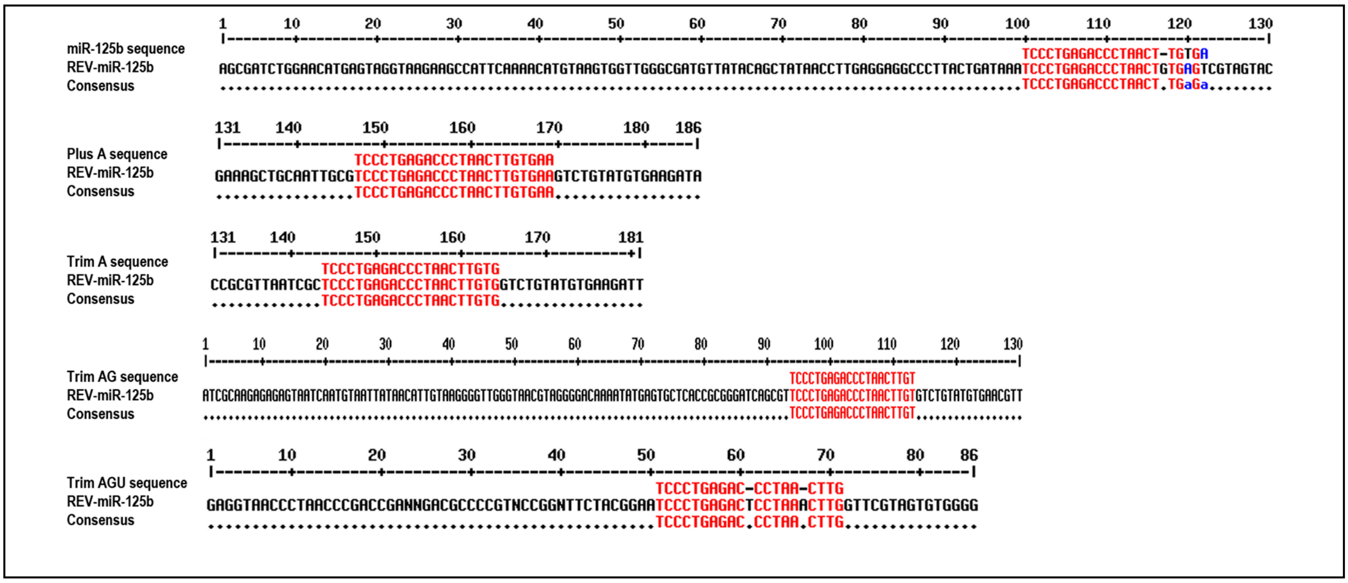


**Suppl. Figure S2:** Sanger sequencing alignment of miR-125b and isomiRs sequences.

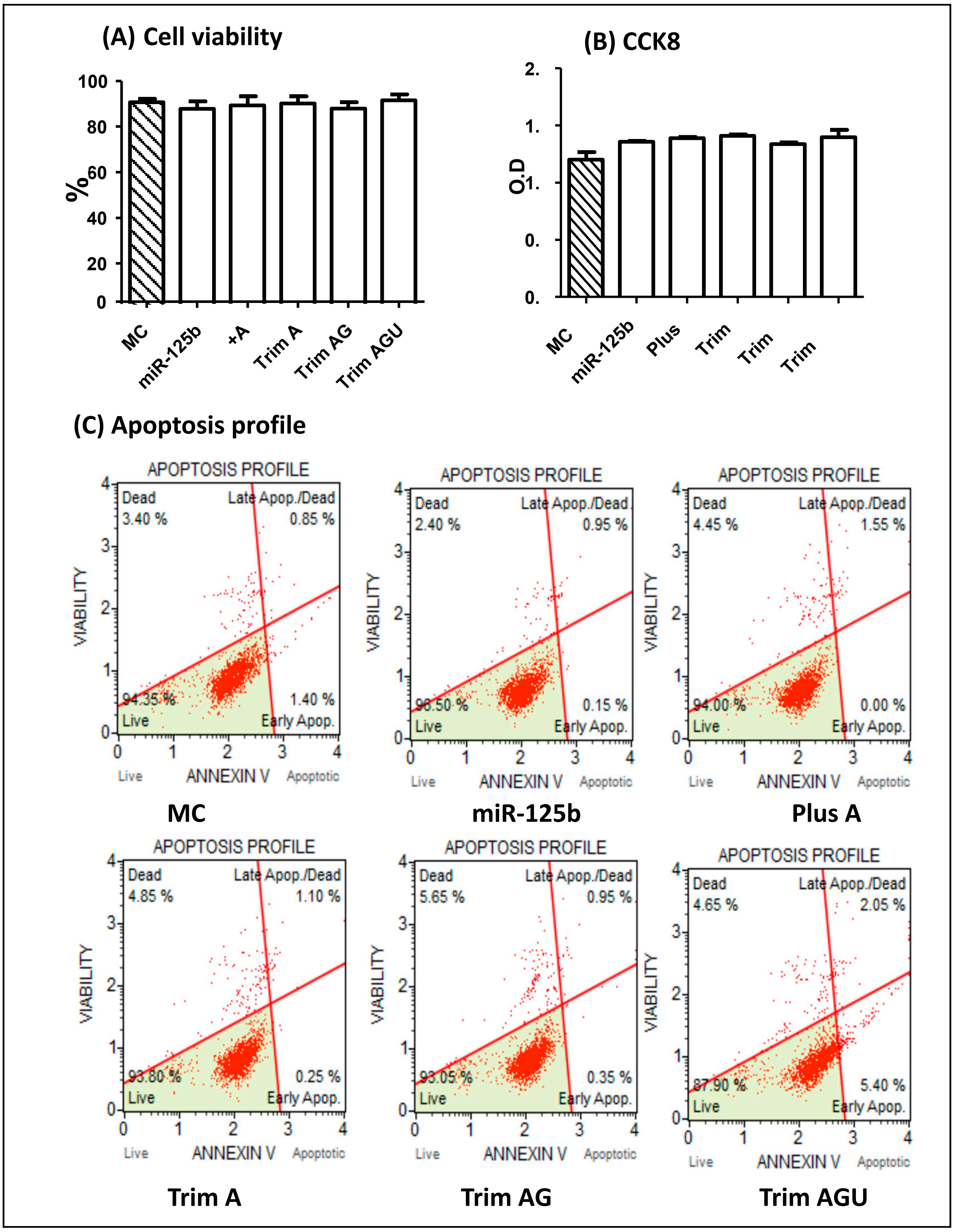


**Suppl. Figure S3:** Effects of miR-125b and its isomiRs under normoxia. miR-125b and its isomiRs mimics were transfected into H9c2. Under normoxia, miR-125b and its isomiRs did not affect the cell viability (A), CCK-8 activity (B) and apoptosis detected by Annexin V/7-AAD double staining (C). Data are presented as Mean ± SD, significance was determined by two-tail unpaired t-test. N=3 in triplicates.

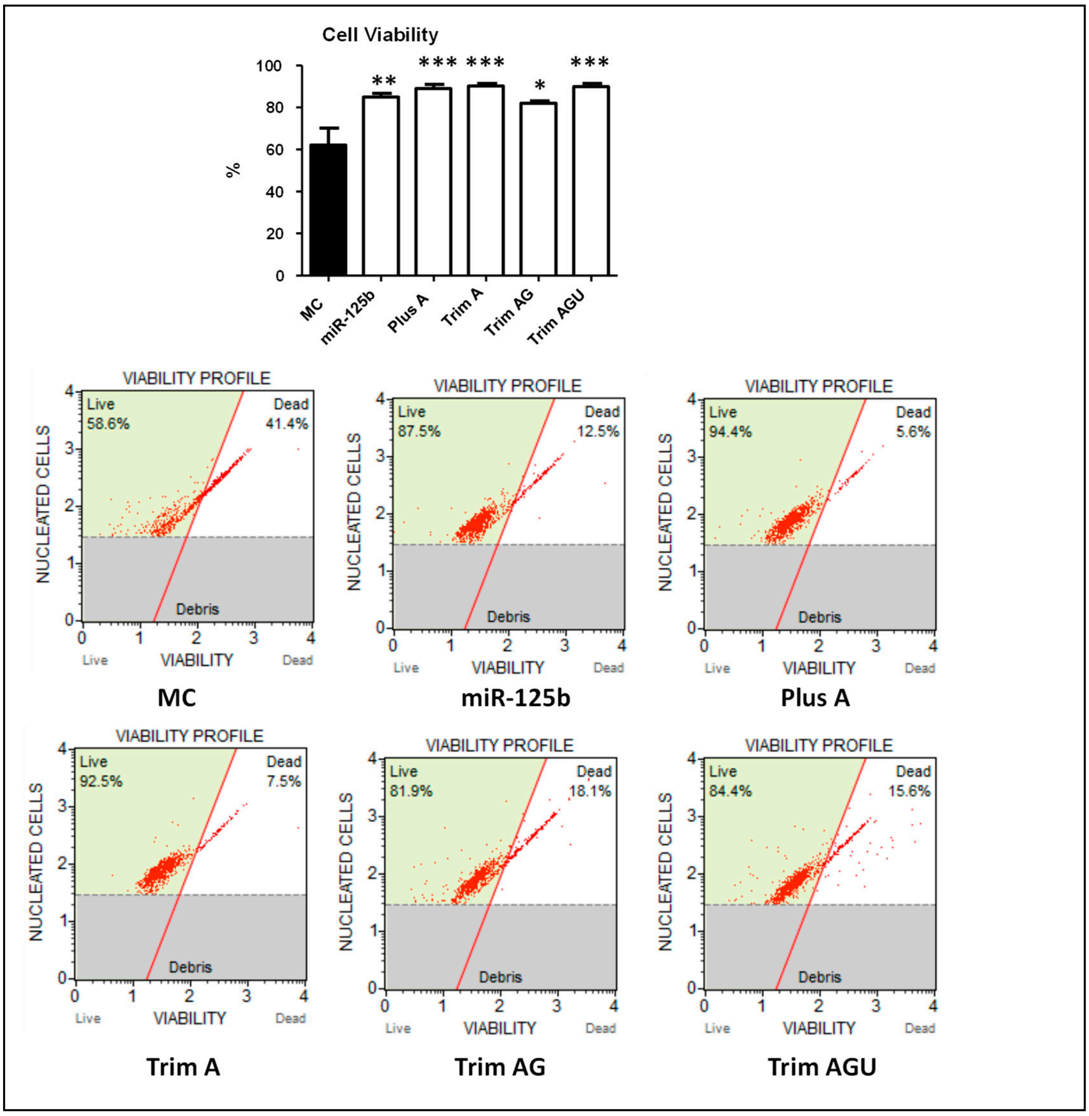


**Suppl Figure S4:** Protective effects of miR-125b and its isomiRs during hypoxia. miR-125b and its isomiRs mimics were transfected into H9c2 and subjected to hypoxia. miR-125b and its isomiRs significantly increased the cell viability as compared with MC. Data are presented as Mean ± SD, *p<0.05, **p<0.01, ***p < 0.001 vs. MC by two-tail unpaired t-test. N=3 in triplicates.

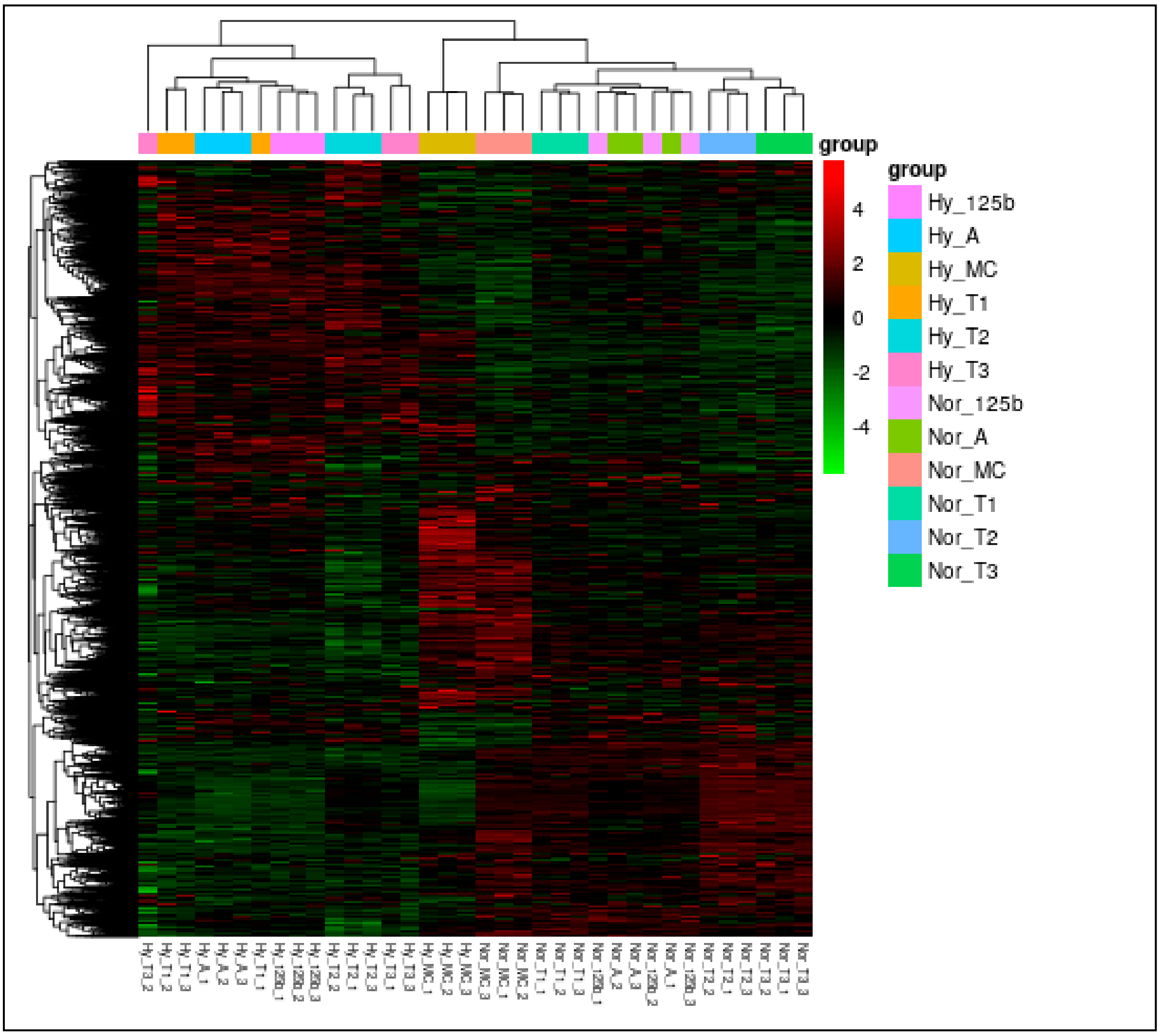


**Suppl Figure S5:** Hierarchical clustering heat map of gene expression for individual samples representing experimental replicates. Nor: Normoxia, Hy: Hypoxia, T1: Trim A, T2: Trim AG and T3: Trim AGU), N=3.

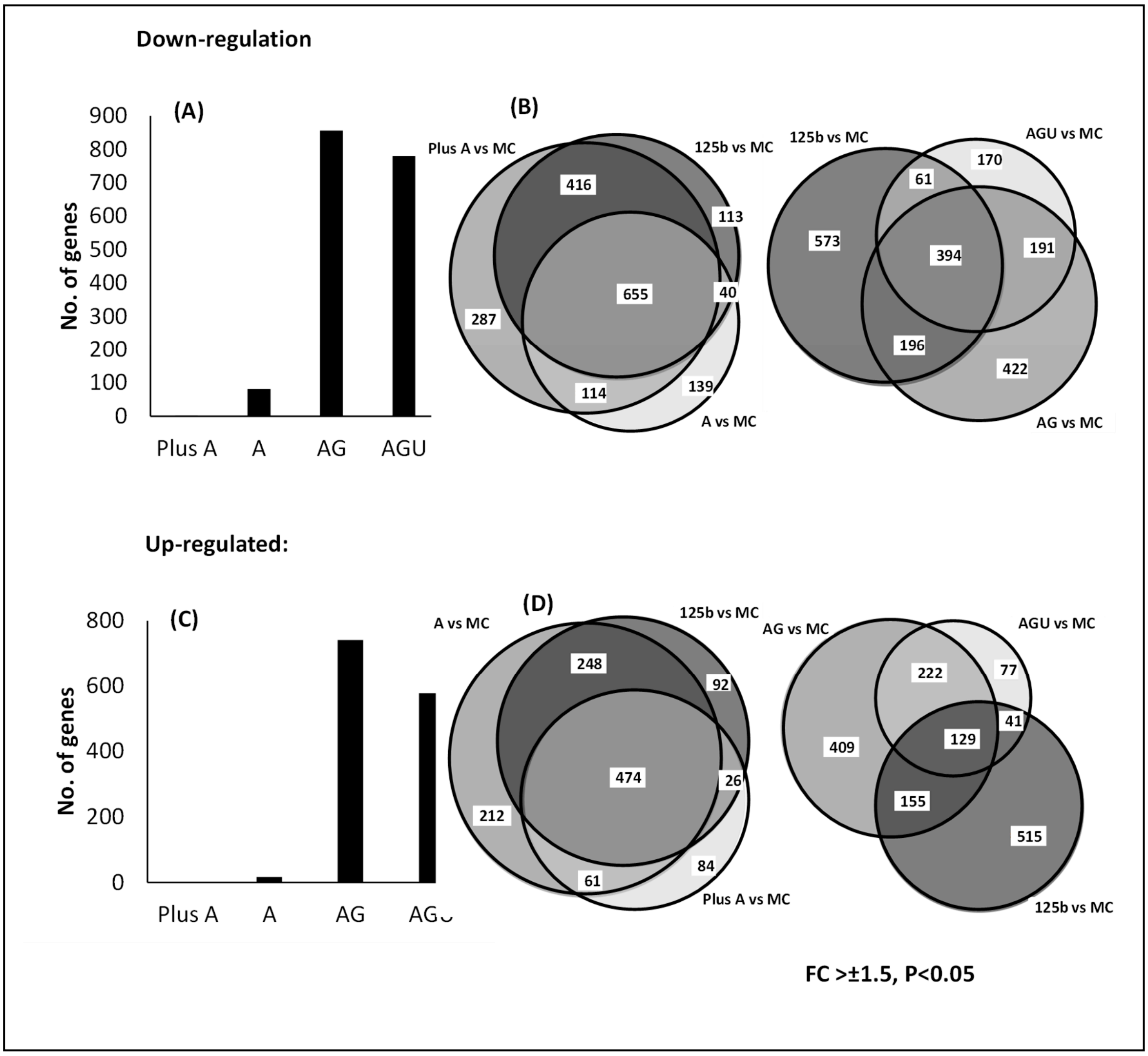


**Suppl Figure S6:** Differences of gene regulation by isomiRs in comparison between miR-125b and its isomiRs under normoxia. Genes regulated by miR-125b and isomiRs by ≥1.5-fold change with p(adj) <0.05 compared to MC were included for further analysis. (A) and (C) show the number of up- and down-regulated genes by direct comparison between miR-125b and isomiRs. The number of overlapping genes between miR-125b and its isomiRs are presented in the proportional venn diagram (B and D).

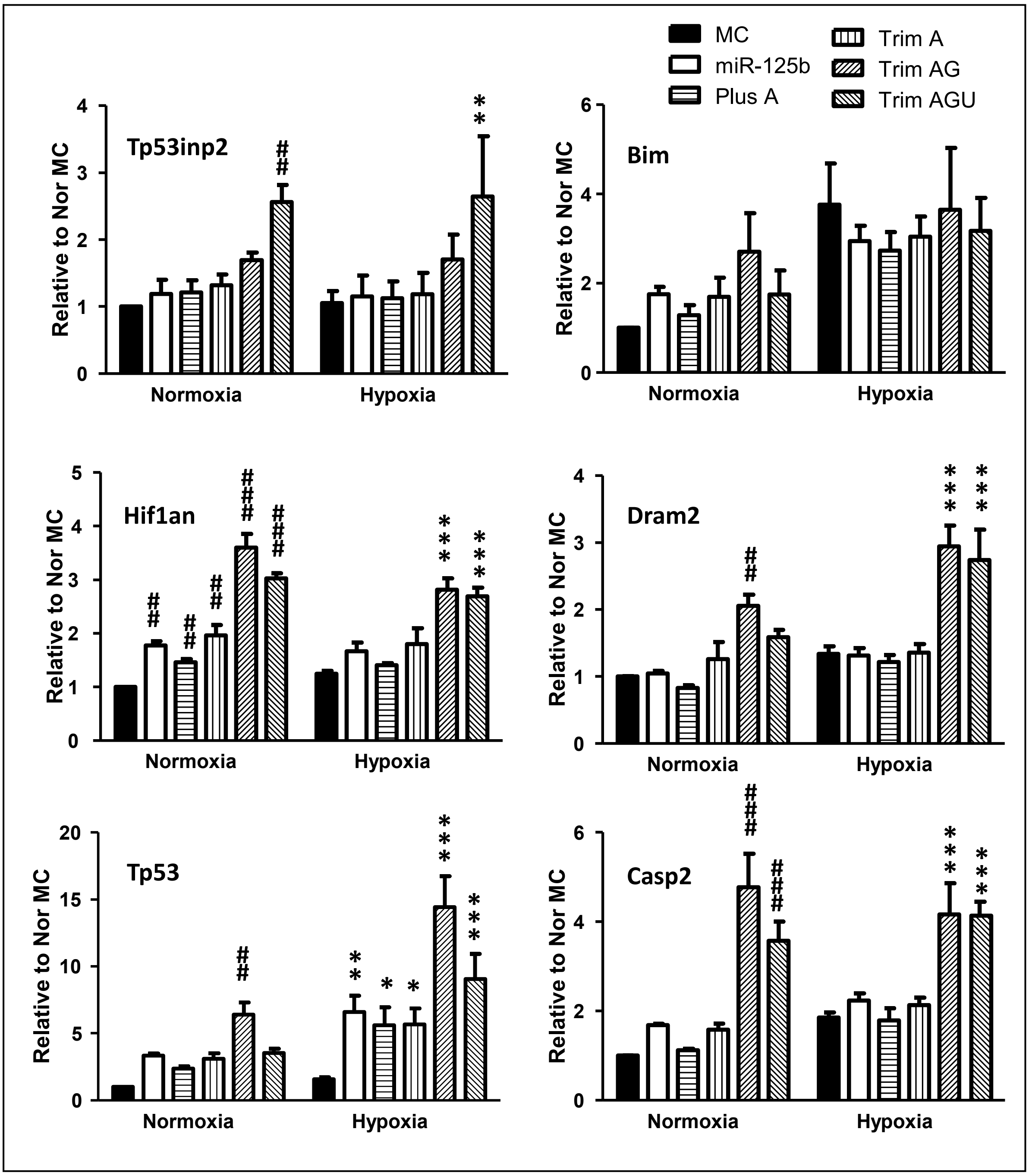


**Suppl Figure S7:** Gene regulations under normoxia and hypoxia assessed by RT-qPCR. Data are presented as Mean ± SD with Student's t-test, * $p < 0.05$, ** $p < 0.01$, ***$p < 0.001$ vs. MC by two-tail unpaired t-test. N=3 in triplicates.
